# Molecular mechanism of GylR-mediated regulation of glycerol metabolism in *Streptomyces clavuligerus* NRRL 3585

**DOI:** 10.3389/fmicb.2022.1078293

**Published:** 2022-11-25

**Authors:** Chaobo Zhang, Youbao Zhao, Zilong Li, Weishan Wang, Ying Huang, Guohui Pan, Keqiang Fan

**Affiliations:** ^1^State Key Laboratory of Microbial Resources, Institute of Microbiology, Chinese Academy of Sciences, Beijing, China; ^2^University of Chinese Academy of Sciences, Beijing, China

**Keywords:** glycerol, metabolism, regulatory mechanism, sequence motif, *Streptomyces*, *Actinobacteria*

## Abstract

Glycerol is a readily available and low-cost simple polyol compound, which can be used as a carbon source for microorganisms to produce various value-added products. Understanding the underlying regulatory mechanism in glycerol metabolism is critical for making better use of glycerol for diverse applications. In a few reported *Streptomyces* strains, the glycerol utilization gene cluster (*glp* operon) was shown to be regulated by the IclR family transcriptional regulator GylR. However, the molecular regulatory mechanism mediated by GylR has not been fully elucidated. In this study, we first analyzed the available *Actinobacteria* genomes in the NCBI Genome database, and found that the *glp* operon-like gene clusters are conserved in *Streptomyces* and several other genera of *Actinobacteria*. By taking *Streptomyces clavuligerus* NRRL 3585 as a model system, we identified that GylR represses the expressions of *glp* operon and *gylR* by directly binding to their promoter regions. Both glycerol-3-phosphate and dihydroxyacetone phosphate can induce the dissociation of GylR from its binding sequences. Furthermore, we identified a minimal essential operator site (a palindromic 18-bp sequence) of GylR-like regulators in *Streptomyces*. Our study for the first time reported the binding sequences and effector molecules of GylR-like proteins in *Streptomyces*. The molecular regulatory mechanism mediated by GylR presumably exists widely in *Streptomyces*. Our findings would facilitate the design of glycerol utilization pathways for producing valuable products. Moreover, our study provided new basic elements for the development of glycerol-inducible regulatory tools for synthetic biology research in the future.

## Introduction

Glycerol is a simple polyol compound existing widely in biological systems (Klein et al., 2017; Doi, 2019). In addition, glycerol is the main by-product of biodiesel production (da Silva et al., 2009; Poblete-Castro et al., 2020). These facts make glycerol as a relatively cheap and readily available substance. Glycerol has a number of industrial applications. It has been widely used in cosmetics and food industry due to its hygroscopic and other properties (Doi, 2019; Poblete-Castro et al., 2020). Furthermore, glycerol is an important substrate in biological industries as it can be converted into various value-added products, such as pharmaceuticals, resins, detergents, plastics and tobacco (Pagliaro et al., 2007; Poblete-Castro et al., 2020). In bacteria, glycerol can be metabolized mainly through two routes (Lin, 1976; Doi, 2019; Poblete-Castro et al., 2020). The first route involves the phosphorylation of glycerol to form glycerol-3-phosphate (G3P), and the subsequent oxidation of G3P to generate dihydroxyacetone phosphate (DHAP). In the other route, glycerol is firstly oxidized to dihydroxyacetone, which is then phosphorylated to afford DHAP. DHAP can be further channeled to downstream metabolic pathways like the glycolysis pathway. Thus it can be seen that glycerol can serve as a common precursor to biosynthesize various products. Usually, the genes encoding the enzymes for conversion of glycerol to DHAP are tightly controlled by different types of transcriptional regulators (Lin, 1976; Hindle and Smith, 1994; Bong et al., 2019; Poblete-Castro et al., 2020). Understanding the molecular regulatory mechanism of glycerol metabolism is critical for better use of glycerol to produce valuable products.

**Figure 1:**
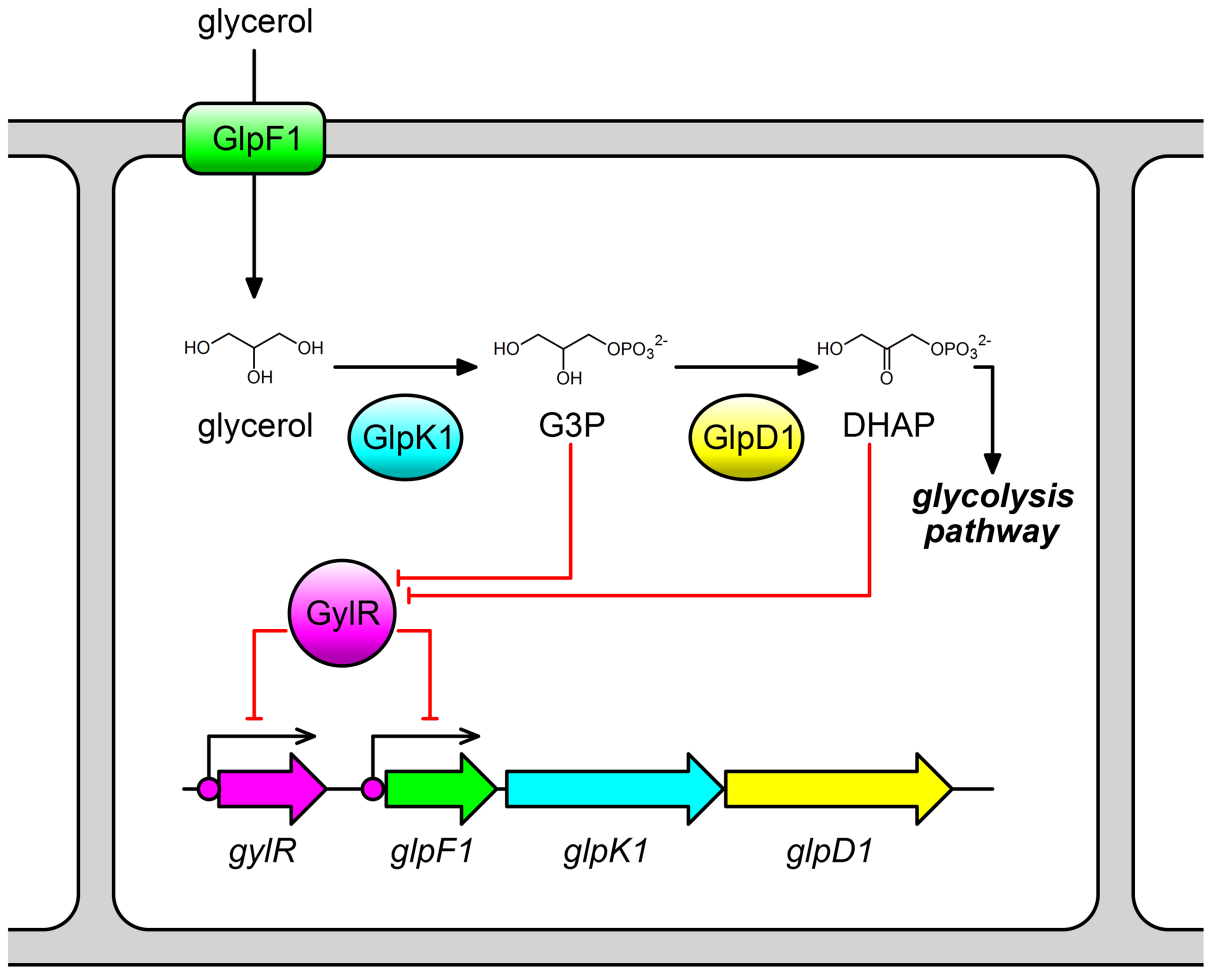
Schematic representation of GylR-mediated regulation on glycerol metabolism in *Streptomyces clavuligerus* NRRL 3585. The *glp* operon consisting of *glpF1*, *glpK1*, and *glpD1*, is negatively regulated by GylR. G3P and DHAP act as effector molecules that can bind to GylR and induce the dissociation of GylR from its binding sites. G3P, glycerol-3-phosphate; DHAP, dihydroxyacetone phosphate.

Streptomycetes are Gram-positive filamentous soil bacteria and are well known as prolific producers of a wide variety of natural products, including many antibacterial, anticancer, and immunosuppressive drugs (Kormanec et al., 2019; Lee et al., 2021). Glycerol can be used as a carbon source by streptomycetes to produce those valuable products, such as the clinically used beta-lactamase inhibitor clavulanic acid produced by *Streptomyces clavuligerus* NRRL 3585 (Saudagar et al., 2008; Guo et al., 2013; Shin et al., 2021). Until now, studies on the glycerol metabolism in streptomycetes have only been reported for *Streptomyces coelicolor* A3(2) (Smith and Chater, 1988a,b; Hindle and Smith, 1994; Lee et al., 2017) and *S. clavuligerus* NRRL 3585 (Baños et al., 2009), both of which contain a glycerol utilization gene cluster. In *S*. *coelicolor* A3(2), the glycerol utilization gene cluster consists of *gylR* and *gylCAB*, in which *gylCAB* corresponds to the *glp* operon (*glpF1K1D1*) in *S. clavuligerus* NRRL 3585 (Smith and Chater, 1988a,b; Baños et al., 2009). The three key glycerol utilization genes (*gylC/glpF1*, *gylA/glpK1*, and *gylB/glpD1*) encode a glycerol transporter, a glycerol kinase, and a FAD-dependent glycerol-3-phosphate dehydrogenase, respectively (Figure 1). Specifically, the transmembrane protein GylC/GlpF1 facilitates the uptake of glycerol into the cells, and then intracellular glycerol is phosphorylated by the kinase GylA/GlpK1 to form G3P, which is subsequently oxidized by the dehydrogenase GylB/GlpD1 to afford DHAP (Figure 1; Baños et al., 2009). Studies in *S*. *coelicolor* A3(2) suggested that GylR negatively regulates the transcription of the *glp* operon. G3P was proposed to be a true effector molecule of GylR, but there is a lack of experimental evidence (Seno and Chater, 1983; Seno et al., 1984). In *S. clavuligerus* NRRL 3585, similar results were observed. The *glp* operon is negatively regulated by GylR. As a member of the IclR family transcriptional regulators, GylR can directly bind to the promoter region of *glp* operon (Figure 1; Baños et al., 2009). *In vivo* studies have revealed that the expression of *glp* operon can be induced by the addition of glycerol during fermentation (Baños et al., 2009; Guo et al., 2013). Studies in non-*Streptomyces* strains, such as *Mycolicibacterium smegmatis* (previously known as *Mycobacterium smegmatis*), indicated that G3P rather than glycerol might bind to GylR-like proteins, and, as a result, regulate the *glp* operon (Bong et al., 2019). Despite the aforementioned studies, the exact binding sequences and the small molecule effectors of GylR-like proteins remain elusive in *Streptomyces*.

In this study, we first carried out a systematic bioinformatic analysis, which revealed that the *glp* operon is highly conserved in *Streptomyces* genomes, and the *glp* operon-like glycerol utilization gene clusters (minimally containing one set of clustered *glpF*, *glpK*, and *glpD* homologous genes) are present in many genera of the phylum *Actinobacteria*. The results implied a common metabolic pathway of glycerol in *Streptomyces*. Then, we took *S. clavuligerus* NRRL 3585 as a model system to further investigate the molecular mechanism of GylR-mediated regulation of glycerol metabolism. Our results showed that GylR negatively regulates the transcriptions of *gylR* and *glp* operon by directly binding to their promoter regions, and the exact binding sequences were identified to contain a conserved 18-bp sequence motif. In addition to G3P, DHAP was found to be another effector molecule that could cause the dissociation of GylR from its binding sequences. Our study for the first time identified the binding sequences and effector molecules of GylR-like proteins in *Streptomyces*, which further elucidated the molecular mechanism of GylR-mediated regulation of glycerol metabolism. The results would facilitate the design of glycerol utilization pathways, and provide valuable regulatory elements for synthetic biology research.

## Materials and methods

### Strains, plasmids, primers, and growth conditions

Bacterial strains and plasmids used in this study are listed in Supplementary Table 1 in the Supplementary material. Primers in this study are listed in Supplementary Table 2. *Escherichia coli* DH5α was used as a general host for propagating plasmids. *E. coli* ET12567 (pUZ8002) (Kieser et al., 2000) was used as a host for transferring DNA from *E. coli* to *Streptomyces* in intergeneric conjugation experiments. *E. coli* Rosetta (DE3) was used as a host for production of GylR protein. *S. clavuligerus* NRRL 3585 and its derived strains were grown on yeast extract-dextrose (YD) agar medium at 28°C (Xiang et al., 2009). The SA medium used for fermentation and RNA extraction is composed of 10 g/l soluble starch, 2 g/l L-asparagine, 21 g/l MOPS, 0.6 g/l MgSO_4_·7H_2_O, and 0.1% (v/v) trace element solution (FeSO_4_·7H_2_O, 1 mg/ml; MnCl_2_·H_2_O, 1 mg/ml; ZnSO_4_·7H_2_O, 1 mg/ml; CaCl_2_·2H_2_O, 1.3 mg/ml; Guo et al., 2013). Antibiotics were used at the following concentrations unless otherwise specified: apramycin, 100 μg/ml; kanamycin, 50 μg/ml; chloramphenicol, 12.5 μg/ml; nalidixic acid, 25 μg/ml. General procedures for *E. coli* and *Streptomyces* manipulations were performed according to the standard protocols (Kieser et al., 2000).

### Bioinformatic analyses

The completed genomes of the phylum *Actinobacteria* were downloaded from the NCBI Genome database (as of September 2022). The *glpF*, *glpK*, *glpD*, and *gylR* homologs were identified using standalone BLASTP program (Camacho et al., 2009) with the previously reported glycerol utilization genes in *S. clavuligerus* NRRL 3585 as query sequences (Baños et al., 2009). Protein sequence comparison was performed using EMBOSS 6.5.0 (Rice et al., 2000). DNA motif search and the data analysis were preformed using Python scripts. The conserved sequence motif logo was generated using MEME Suite 5.5.0 (Bailey et al., 2006). The information of all the identified homologs, including their GenBank accession numbers and sequences, was provided in Supplementary Sheet 1. The information of all the clustered homologs was provided in Supplementary Sheet 2. The information of all the DNA sequences corresponding to the conserved 18-bp sequence motif was provided in Supplementary Sheet 3.

### Construction of the mutant strain *Streptomyces clavuligerus ΔgylR*

To construct a plasmid for inactivation of *gylR*, a 1,461-bp upstream DNA fragment was amplified with primers gylR-U-F and gylR-U-R, and a 1,469-bp downstream DNA fragment was amplified with primers gylR-D-F and gylR-D-R, using *S. clavuligerus* NRRL 3585 genomic DNA as the template. After digestion with appropriate enzymes (*Eco*RI and *Bam*HI for upstream fragment, *Bam*HI and *Hin*dIII for downstream fragment), the two fragments were inserted into *Eco*RI/*Hin*dIII-digested pKC1139 to generate pKC1139-gylR, which was verified by DNA sequencing. The plasmid pKC1139-gylR was transformed into *E. coli* ET12567 (pUZ8002) and then introduced into *S. clavuligerus* NRRL 3585 by intergeneric conjugation. After several rounds of passaging the exconjugants on solid YD medium, apramycin sensitive colonies were screened by PCR using primers gylR-del-F and gylR-del-R to obtain the double-crossover mutants Δ*gylR* (Supplementary Figure 1).

### RNA extraction and quantitative reverse transcription PCR

The transcriptional levels of the analyzed genes in *S. clavuligerus* NRRL 3585 and Δ*gylR* mutant strains were evaluated by quantitative reverse transcription PCR (RT-qPCR). Total RNAs were isolated from the cultures grown in SA media without or with 163 mM glycerol at 24 h as in previous studies (Baños et al., 2009; Guo et al., 2013). The RNA samples were treated with RQ1 RNase-free DNase (Promega) to remove genomic DNA. Synthesis of cDNA and subsequent RT-qPCR were performed according to the standard methods (Li et al., 2015), and transcription of the *hrdB* gene was used as the internal control.

### Promoter activity assay

The green fluorescent protein (GFP) gene was amplified by PCR from pIJ-Potr using primer pair GFP-F and GFP-R (Wang et al., 2016). The PCR product was digested with *Spe*I and *Eco*RI, and then inserted into the corresponding sites of the integrative plasmid pSET152 to generate pSET152-GFP. The promoter region of *glp* operon (P*glp*) was amplified with primer pair Pglp-F and Pglp-R using *S. clavuligerus* NRRL 3585 genomic DNA as the template. The PCR product was digested with *Bam*HI and *Spe*I, and then inserted into pSET152-GFP. The resultant plasmid pSET152-Pglp-GFP was introduced into *S. clavuligerus* NRRL 3585 and Δ*gylR* mutant strains through conjugation to obtain WT-Pglp-GFP and Δ*gylR*-Pglp-GFP, respectively. As a control, the plasmid pSET152-GFP was transformed into *S. clavuligerus* NRRL 3585 to generate the recombinant strain WT-GFP. The above recombinant strains were cultivated in SA medium for 12 h. Then, 200 μl of cell culture was washed twice with pure water, and transferred into 96-well plates. The fluorescence intensities were recorded using a Synergy^™^ HT Multi-Mode Microplate Reader with an excitation at 489 nm and an emission at 512 nm for analysis. Pure water was used as a blank control. The biomass of *S. clavuligerus* strains were detected by the simplified diphenylamine colorimetric method (Zhao et al., 2013).

### Overproduction and purification of GylR

The coding region of *gylR* was amplified by PCR from *S. clavuligerus* NRRL 3585 genomic DNA using primer pair gylR-F and gylR-R. The PCR product was digested with *Nde*I and *Xho*I and then inserted into the corresponding sites of pET-30a. The resultant plasmid pET30a-gylR was verified by DNA sequencing and subsequently introduced into *E. coli* Rosetta (DE3) for production of GylR. The *E. coli* Rosetta (DE3) harboring pET30a-gylR was cultured in LB supplemented with 50 μg/ml kanamycin at 37°C and 250 rpm until an OD_600_ of 0.6 was reached, at which time point 0.1 mM isopropyl-β-D-thiogalactopyranoside (IPTG) was added, and the strain culture was allowed to incubate at 30°C for 4 h. The cells were harvested, and resuspended in lysis buffer (20 mM Tris–HCl, pH 8.0, 500 mM NaCl, 5 mM imidazole, and 10% sorbitol).

The C-terminal His_6_-tagged GylR was purified using nickel-nitrilotriacetic acid (Ni-NTA) agarose chromatography according to the manufacturer’s protocol (Novagen) with glycerol in all the buffers being substituted by sorbitol. Of note, the elution buffer used for the purification of GylR contained 500 mM imidazole and 10% (w/v) sorbitol. As purified GylR was unstable and lost activity quickly, we used freshly purified GylR in all the assays in this study. The protein concentration was determined by using a bicinchoninic acid (BCA) protein assay kit (Novagen).

### Size-exclusion chromatography assays

The polymerization state of GylR was analyzed by size-exclusion chromatography on a Shimadzu Prominence HPLC system using TSKgel G3000 SWxl column (7.8 × 300 mm, TOSOH Inc.) with an elution solvent of 0.1 M Na_2_SO_4_ (pH 6.7) and a flow rate of 0.5 ml/min as described previously (Pan et al., 2017). Bovine serum albumin, ovalbumin, and AlpJ were used as molecular weight standards.

### Electrophoretic mobility shift assays

The electrophoretic mobility shift assays (EMSAs) were performed according to the protocol described previously (Wang et al., 2009). The DNA probes BS1 (upstream region of the coding sequence of *gylR*) and BS2 (upstream region of the coding sequence of *glpF1*) were amplified from *S. clavuligerus* NRRL 3585 genomic DNA with primer pairs BS1-F/BS1-R, and BS2-F/BS2-R, respectively. Other DNA probes were synthesized by Invitrogen. A typical 20 μl reaction mixture consisted of 10 ng/μl DNA probe, GylR (various concentrations), small molecules (glycerol or G3P or DHAP with different concentrations), 50 mM HEPES (pH 7.5), 1 mM dithiothreitol (DTT), 5 mM MgCl_2_, 0.2 mg/ml bovine serum albumin (BSA), 2 mM EDTA and 5% sorbitol. After incubation at 25°C for 25 min, protein-bound DNA and free DNA were separated by electrophoresis on nondenaturing 6.0% (w/v) polyacrylamide gels with a running buffer (pH 7.5) containing 200 mM HEPES and 4 mM EDTA. Gels were stained with SYBR Gold. The concentrations of DNA probes were determined with a NanoVue Plus spectrophotometer.

### DNase I footprinting assay

In order to determine the binding sites of GylR, labeled BS1 and BS2 probes were amplified by PCR with primer pairs FAM-gylR/HEX-gylR and FAM-glpF1/HEX-glpF1, respectively. A typical 50 μl reaction mixture contained 200 ng FAM/HEX-labeled DNA probe, 0–40 ng/μl GylR, 100 mM KCl, 20 mM HEPES (pH 7.5), 2 mM DTT, 5 mM MgCl_2_, 0.5 mg/ml BSA, and 5% sorbitol. After incubation at 25°C for 25 min, 5.5 μl RQ1 RNase-free DNase I buffer and 0.375 U DNase I were added. The mixture was incubated at 25°C for an additional 80 s. Then, the reaction was stopped by addition of 10 μl stop solution (20 mM EGTA, pH 8.0) and 100 μl phenol-chloroform (1:1, v/v). Samples were analyzed by Genolab Biotech Co., Ltd.

### Rapid amplification of 5′ cDNA ends (5′-RACE)

The transcription start points (tsp) of *gylR*, and *glp* operon were determined by using a FirstChoice RLM-RACE kit (Ambion) following the manufacturer’s instructions. Total RNAs from *S. clavuligerus* NRRL 3585 were prepared as described above (Guo et al., 2013). Total RNAs (10 ng) were successively treated with calf intestine alkaline phosphatase (CIP), tobacco acid pyrophosphatase (TAP) and ligated with a 45-base 5′-RACE adapter at the 5′-end using T4 RNA ligase. Reverse transcription was performed using SuperScript III reverse transcriptase with the supplement of random decamers. PCR was first performed using a 5’-RACE outer primer and gene-specific outer primer with *Q5* DNA polymerase (New England Biolabs). This PCR product was used as the template of inner PCR using a 5′-RACE inner primer and a gene-specific inner primer. A 25 μl volume of each PCR reaction mixture was analyzed by 2% agarose gel electrophoresis. The product of inner PCR was sent for sequencing after gel purification.

## Results

### The distribution of *glp* operon-like glycerol utilization gene clusters in *Actinobacteria*

**Figure 2:**
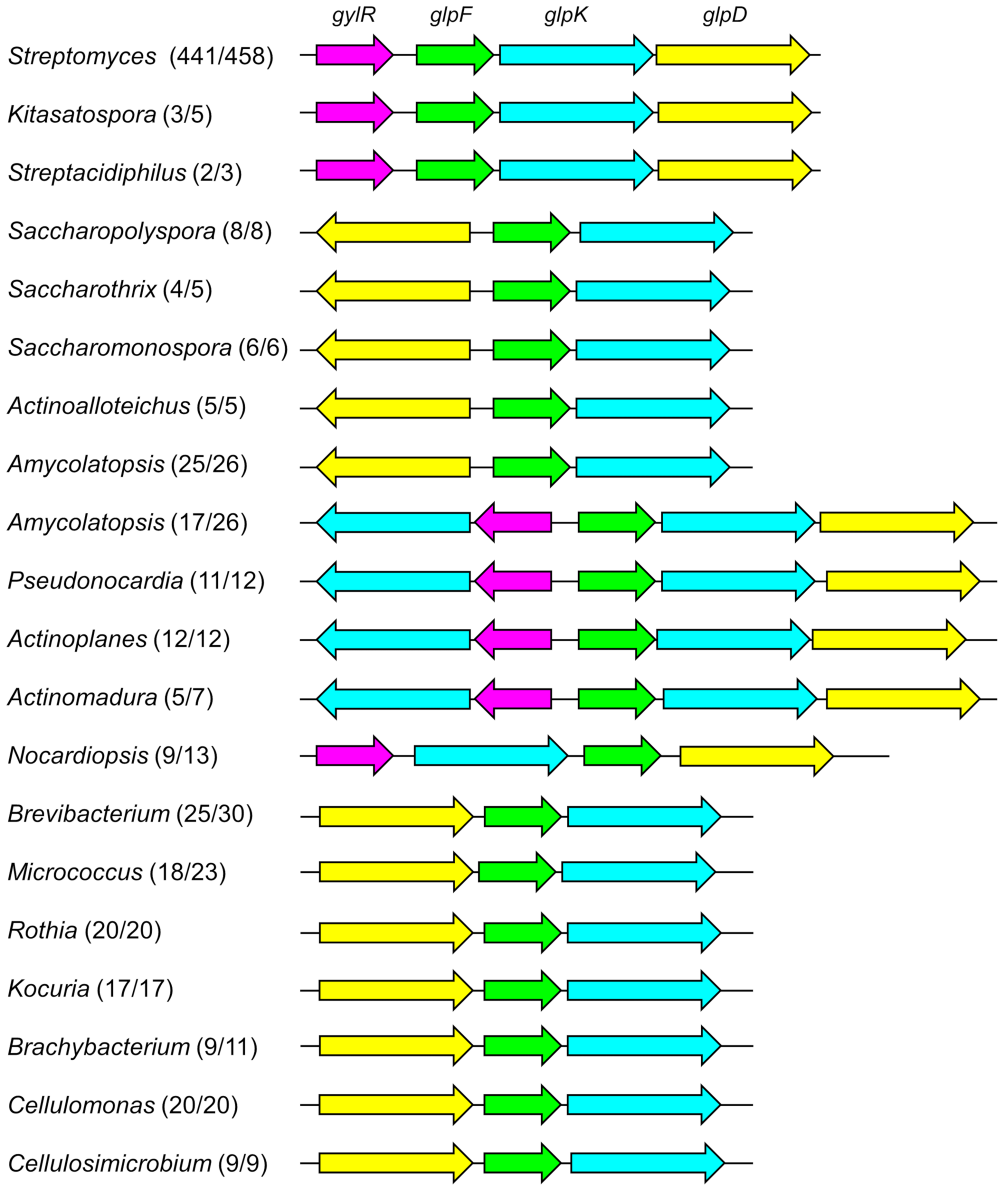
The distribution of *glp* operon-like gene clusters in analyzed genomes of the phylum *Actinobacteria*. The numbers after the name of each genus represented the frequency of *glp* operon-like gene clusters being identified, e.g., *Streptomyces* (441/458) denoted that 441 out 458 *Streptomyces* genomes each contains a *glp* operon-like gene cluster. The genera, in which less than 50% genomes harbor the *glp* operon-like gene clusters, were not included in this figure.

Previous studies strongly suggested that *glp* operon probably serves as a general pathway in glycerol metabolism in *Streptomyces* strains (Smith and Chater, 1988b; Hindle and Smith, 1994; Baños et al., 2009). Indeed, we found that the *glp* operon accompanied by *gylR* is highly conserved in the genus *Streptomyces*, as 441 out of 458 completed genomes of *Streptomyces* in the NCBI Genome database (as of Sep. 2022) each harbor a *glp* operon with an adjacent *gylR* homologous gene (Figure 2; Supplementary Sheets 1, 2). This encouraged us to further analyze the distribution of *glp* operon-like gene clusters (minimally containing one set of clustered *glpF*, *glpK*, and *glpD* homologous genes) in other genera of the phylum *Actinobacteria*. Therefore, we performed a systematic analysis of the remaining 3,112 completed genomes of *Actinobacteria* available in the NCBI Genome database (as of Sep. 2022). The *glpF*, *glpK*, *glpD*, and *gylR* homologous genes identified in each of the *Actinobacteria* genomes were shown in Supplementary Sheet 1. Overall, 1,517 out of 3,570 genomes each contain at least one set of *glpF*, *glpK*, and *glpD* homologous genes (not necessarily clustered). The bioinformatic analysis showed that the *glp* operon-like gene clusters often exist in *Kitasatospora* and *Streptacidiphilus*, the other two genera of the family Streptomycetaceae (Figure 2; Supplementary Sheet 2). In several genera of the family Pseudonocardiaceae, including *Saccharopolyspora*, *Saccharothrix*, *Saccharopolyspora*, *Actinoalloteichus*, and *Amycolatopsis*, almost every genome of them contains a gene cluster consisting of *glpFK* homologous genes, and an upstream *glpD* homolog (transcribed in the opposite direction). However, no *gylR* homolog was found around those gene clusters. Interestingly, over half of the *Amycolatopsis* genomes also contain another gene cluster. In each of these gene clusters, a *gylR* homolog and another *glpK* homolog are present upstream of the *glpFKD* homologous genes. Such a gene cluster is present in most of the genomes of *Pseudonocardia*, *Actinoplanes*, and *Actinomadura*. In the majority of *Nocardiopsis* genomes, a *gylR* homolog and *glpKFD* homologous genes were found clustered together. In most genomes of *Brevibacterium*, *Micrococcus*, *Rothia*, *Kocuria*, *Brachybacterium*, *Cellulomonas*, and *Cellulosimicrobium*, a gene cluster, in which a *glpD* homolog is located upstream of the *glpFK* homologous genes, was identified without *gylR* homologs nearby. In some other genera, such as *Corynebacterium*, *Rhodococcus*, *Mycolicibacterium*, and *Nocardia*, *glp* operon-like gene clusters were found in less than 50% analyzed genomes (Supplementary Sheet 2). Surprisingly, in the analyzed genomes of *Mycobacterium*, the *glpF*, *glpK* and *glpD* homologous genes were all found to be standalone proteins. It is worth pointing out again that the strain previously called *Mycobacterium smegmatis* (harboring a *glp* operon-like gene cluster) was renamed *Mycolicibacterium smegmatis* based on the updated taxonomy system of NCBI.

Overall, the results showed that *glp* operon-like gene clusters are present in many genera of *Actinobacteria*, and these clusters are likely to play important roles in the glycerol metabolism in *Actinobacteria*. It is obvious that *Actinobacteria* strains have evolved diverse *glp* operon-like gene clusters, which might be beneficial to different host strains for utilizing glycerol.

### GylR represses the expression of *glp* operon and *gylR* itself

**Figure 3:**
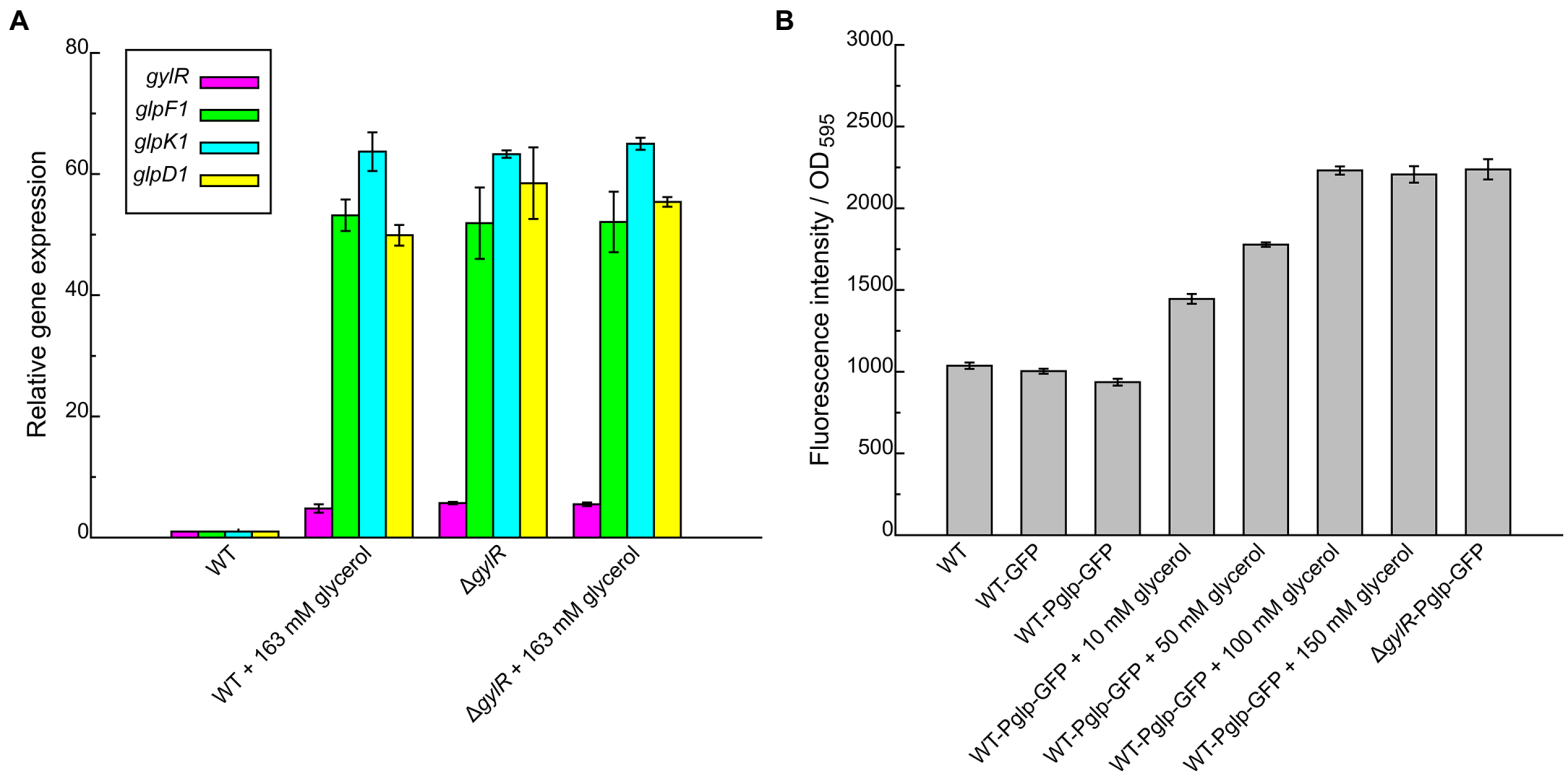
Transcriptional analysis of *gylR* and *glp* operon in *Streptomyces clavuligerus* NRRL 3585 and recombinant strains under different culture conditions. **(A)** RT-qPCR analysis of the transcriptional levels of *gylR*, *glpF1*, *glpK1*, and *glpD1* in *Streptomyces clavuligerus* NRRL 3585 (WT) and Δ*gylR* cultivated in SA medium or SA medium supplemented with 163 mM glycerol. The *hrdB* gene (encoding the RNA polymerase principal sigma factor) was used as an internal control. The relative gene expression values in WT were arbitrarily assigned as 1. **(B)** Evaluation of the activities of P*glp* (promoter region of *glp* operon) in WT and recombinant strains cultivated in SA medium or SA medium supplemented with different concentrations of glycerol. The fluorescence intensity of GFP and biomass (OD_595_) were analyzed after 12 h of cultivation. OD_595_ values were determined using the simplified diphenylamine colorimetric method (Zhao et al., 2013). Error bars represent standard deviations of three independent replicates.

The above bioinformatic analysis further supported that *glp* operon together with *gylR* serves as an important general pathway in glycerol metabolism of *Streptomyces*. Here, we took *S. clavuligerus* NRRL 3585 as a model strain to further investigate the molecular regulatory mechanism of GylR in glycerol metabolism. To better understand the regulatory role of GylR, we generated the mutant strain *S. clavuligerus* Δ*gylR* with a truncation in *gylR* gene (Supplementary Figure 1). For the wild-type (WT) strain NRRL 3585, the transcriptions of *glpF1, glpD1* and *glpK1* were at low levels at the absence of glycerol, while the addition of 163 mM glycerol to the SA medium significantly augmented the transcriptions of *glpF1, glpD1* and *glpK1* (Figure 3A). However, for the mutant strain Δ*gylR*, the transcriptions of the three genes were all at high levels with or without the addition of glycerol. In addition, we found that the transcription of *gylR* was also induced by glycerol in the WT strain, and the transcription of truncated *gylR* in Δ*gylR* was at a similarly high level regardless of whether glycerol was added or not (Figure 3A). These results showed that GylR is a repressor regulating the transcriptions of the *glp* operon, as well as *gylR* itself in *S. clavuligerus*, which was consistent with the previous reports in *S. clavuligerus* (Baños et al., 2009) and *S. coelicolor* (Smith and Chater, 1988b).

Furthermore, we analyzed the activity of P*glp* (a DNA fragment containing the promoter of *glp* operon) using green fluorescent protein (GFP) as a reporter. The reporter plasmid pSET152-Pglp-GFP was constructed and transformed into the WT and Δ*gylR* strains, affording the recombinant strains WT-Pglp-GFP and Δ*gylR*-Pglp-GFP, respectively. A control plasmid pSET152-GFP was also constructed and transformed into WT to obtain the recombinant strain WT-GFP. We observed that, in comparison to that of WT-GFP, the transcription of *glp* operon in WT-Pglp-GFP was induced by glycerol in a concentration-dependent manner, and reached the maximum level when glycerol concentration was above 100 mM (Figure 3B). As expected, the transcription of *glp* operon in Δ*gylR*-Pglp-GFP was at a level comparable to the maximum level in the WT-Pglp-GFP strain induced by glycerol. These results further confirmed that GylR negatively regulates the expression of *glp* operon, and also implied that GylR serves as the main regulator of *glp* operon.

### GylR directly binds to the promoter regions of *glp* operon and *gylR*

**Figure 4:**
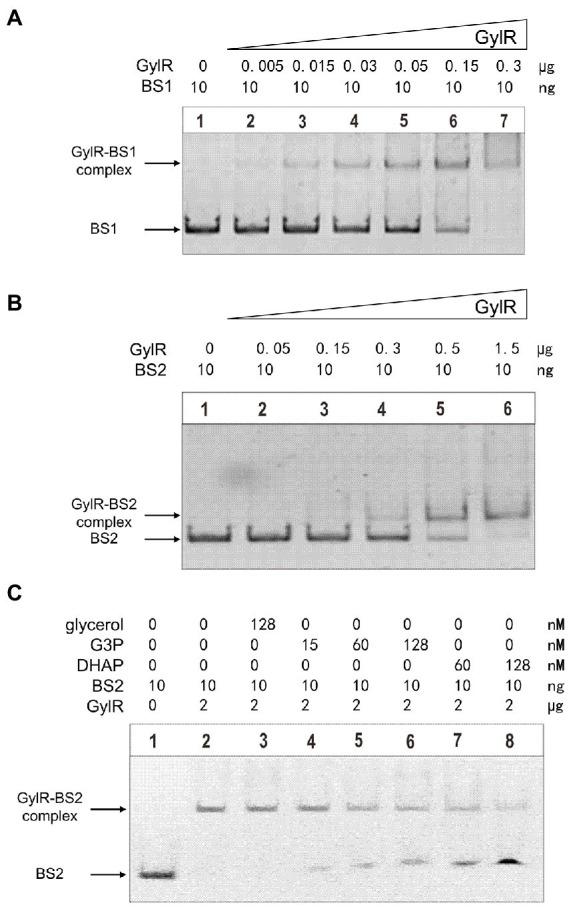
Binding of GylR to the promoter regions of *gylR* and *glp* operon, and identification of the effector molecules of GylR by EMSAs. **(A)** EMSAs of GylR binding to probe BS1 containing P*glp* (promoter of *glp* operon). **(B)** EMSAs of GylR binding to probe BS2 containing P*gyl* (promoter of *gylR*). **(C)** Effects of glycerol, G3P, and DHAP on GylR binding to BS2 by EMSAs.

Although GylR was proposed to regulate the *glp* operon and *gylR* through binding to their promoter regions in *S. clavuligerus*, there is a lack of experimental evidences (Baños et al., 2009). We overproduced C-terminal His_6_-tagged GylR in *E. coli* Rosetta (DE3) and purified it to homogeneity by Ni-NTA affinity chromatography (Supplementary Figure 2A). Size-exclusion chromatography analysis revealed that GylR forms a dimer under the tested conditions (Supplementary Figure 2B), which is reasonable as IclR family transcription factors typically bind to their target sequences as dimers or tetramers (Suvorova and Gelfand, 2021). Then, the electrophoretic mobility shift assay (EMSA) was carried out to determine the direct interactions between GylR and the promoter regions of *gylR* and *glp* operon. Two DNA fragments (BS1 and BS2) containing the promoters of *gylR* and *glp* operon, respectively, were used as DNA probes in the EMSA studies. As shown in Figures 4A,B, GylR could directly bind to both BS1 and BS2 in a concentration dependent manner. Together, the above results strongly supported that GylR negatively regulates the expression of *glp* operon and *gylR* through directly binding to their promoter regions.

### Glycerol-3-phosphate and dihydroxyacetone phosphate are the effector molecules of GylR

To date, the effector molecules that could modulate the binding affinity of GylR to its target DNA regions have not been identified in streptomycetes. Previous preliminary studies in strains like *S. coelicolor* A3(2) and *Mycolicibacterium smegmatis* (Seno et al., 1984; Bong et al., 2019) inspired us to propose that the metabolites in the early stage of glycerol metabolic pathway might serve as the effector molecules of GylR in *S. clavuligerus* NRRL 3585. Therefore, we tested the effects of adding glycerol and its metabolites, G3P and DHAP, to the aforementioned EMSA reactions of GylR with the DNA probe BS2. The results showed that both G3P and DHAP were capable of inducing the dissociation of GylR from BS2, whereas glycerol could not induce the dissociation under tested conditions (Figure 4C). It is worth noting that DHAP was discovered for the first time as an effector molecule of GylR-like regulatory proteins.

### Characterization of the binding sequences of GylR revealing a conserved sequence motif

**Figure 5:**
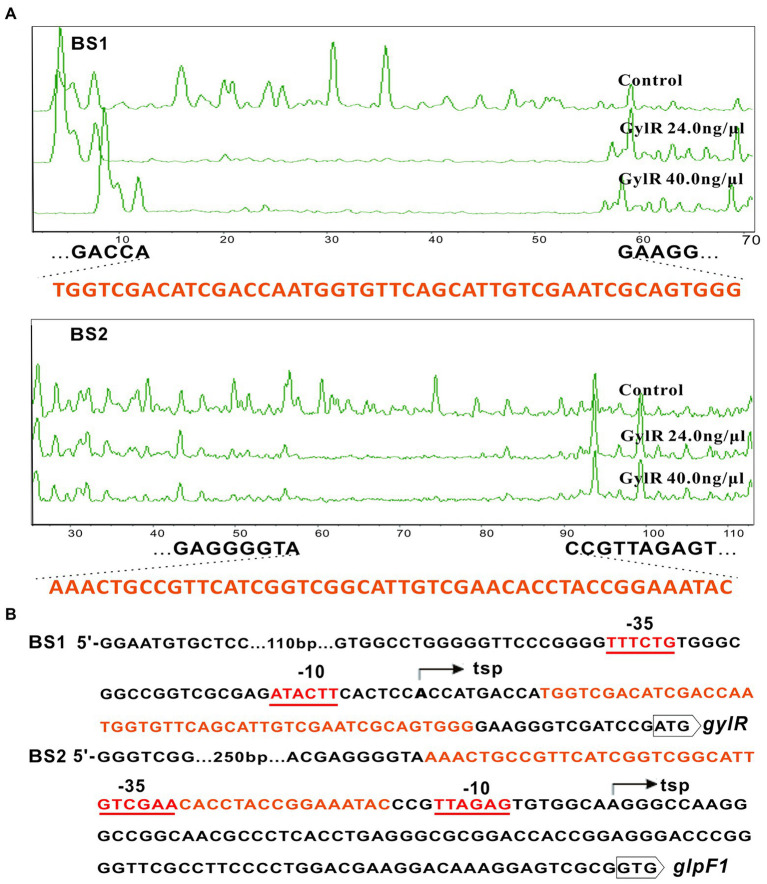
Identification of the binding sequences of GylR and the transcription start sites of *gylR* and *glpF1*. **(A)** DNase I footprinting assays of GylR with the probes BS1 and BS2. Footprints of GylR were shown between dashed lines. **(B)** Sequence analysis of BS1 and BS2. The identified binding sequences of GylR on BS1 and BS2 were shown in orange. The transcription start sites (tsp) of *gylR* and *glpF1* were determined and marked by bent arrows. The putative − 10 and − 35 regions of both promoters P*gyl* and P*glp* were underlined.

To elucidate the exact binding sequences of GylR at the promoter regions of *gylR* and *glp* operon, DNase I footprinting experiments were carried out. The results revealed two protected regions including a region from position +11 to +57 (5′-TGGTCGACATCGACCAATGGTGTTCAGCATTGTCGAATCGCAGTGGG-3′) relative to the transcription start point (tsp) of *gylR*, and the other region from position −17 to −63 (5′-AAACTGCCGTTCATCGGTCGGCATTGTCGAACACCTACCGGAAATAC-3′) relative to the tsp. of the *glp* operon (Figure 5A). As shown in Figure 5B, the binding region upstream of *gylR* is located right after the −10 region of promoter P*gyl*, which implied that GylR represses *gylR* through impeding the transcription elongation. On the other side, the binding region upstream *glpF1* overlaps with the promoter P*glp*, suggesting that GylR inhibits the transcription of *glp* operon by hindering the binding of RNA polymerase to P*glp*.

**Figure 6:**
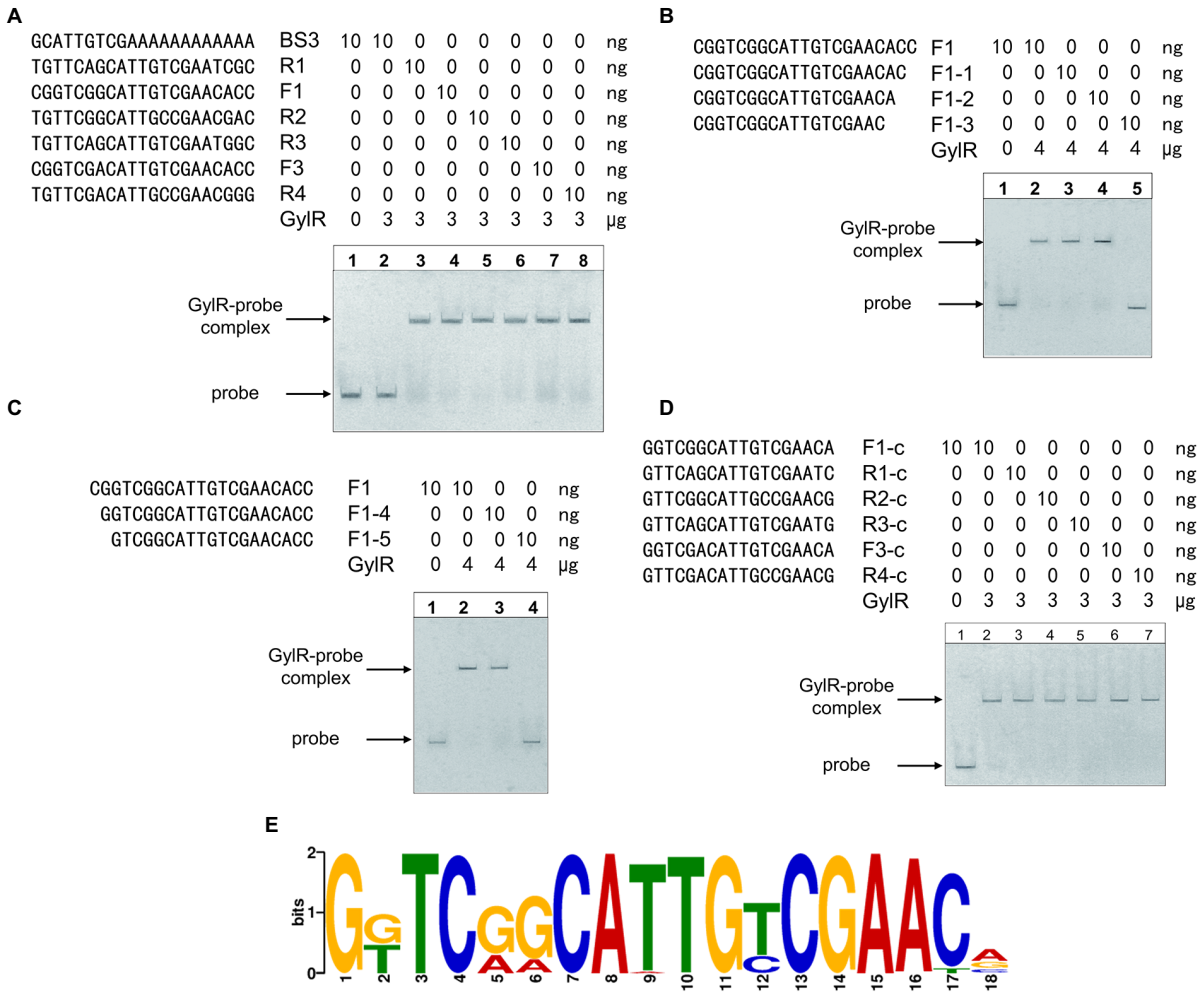
Identification of the core binding sequences of GylR revealing an 18-bp sequence motif. **(A)** EMSAs of GylR binding to the 21-bp DNA sequences from several representative *Streptomyces* strains. The DNA probes R1, R2, R3, and R4 referred to the corresponding 21-bp DNA sequences upstream of *gylR* homologs in *S. clavuligerus* NRRL 3585, *S. coelicolor* A3(2), *S. albus* DSM 41398, and *S. avermitilis* MA-4680, respectively. The DNA probes F1 and F3 referred to the corresponding 21-bp DNA sequences upstream of *glpF1* homologs in *S. clavuligerus* NRRL 3585, and *S. albus* DSM 41398, respectively. The corresponding 21-bp DNA sequences upstream of *glpF1* homologs in *S. coelicolor* A3(2) and *S. avermitilis* MA-4680 were identical to that of probe F1. The probe BS3 was used as a negative control. **(B)** EMSAs of GylR binding to trimmed F1 probes on the 3-prime end. **(C)** EMSAs of GylR binding to trimmed F1 probes on the 5-prime end. **(D)** EMSAs of GylR binding to the core 18-bp DNA sequences derived from the aforementioned representative 21-bp DNA sequences. F1-c, R1-c, R2-c, R3-c, F3-c, and R4-c referred to the core 18-bp DNA sequences derived from the probes F1, R1, R2, R3, F3, and R4, respectively. **(E)** The conserved 18-bp sequence motif bound by GylR-like proteins in *Streptomyces* strains. The 18-bp sequence motif was obtained by analyzing the putative binding sequences upstream of *gylR* homologs and *glpF1* homologs in available *Streptomyces* genomes with MEME Suite 5.5.0 (Bailey et al., 2006).

Analysis of the two binding regions of GylR, and their homologous DNA regions upstream of *gylR* and *glpF1* homologs in representative *Streptomyces* strains including *S. coelicolor* A3(2), *Streptomyces albus* DSM 41398, and *Streptomyces avermitilis* MA-4680, suggested a relatively conserved 21-bp sequence region (5′-YGKTCRRCATTGYCGAAYVVS-3′; Figure 6A). Then, the corresponding 21-bp DNA probes were synthesized and tested in the EMSAs. The results showed that GylR could bind to all these 21-bp DNA probes (Figure 6A). To identify the minimal core DNA sequences that interact with GylR to form a complex, the 21-bp probe F1 (derived from BS2) was trimmed from two ends separately to afford a set of shortened DNA probes, which were tested in the subsequent EMSAs (Figures 6B,C). The results allowed us to narrow down to a minimum 18-bp palindromic DNA sequence F1-c (5′-GGTCGGCATTGTCGAACA-3′), which could be bound by GylR (Figure 6D). Then, the aforementioned 21-bp DNA probes were all shortened to 18-bp DNA probes, and the EMSA results showed that GylR was able to bind all these 18-bp DNA sequences (Figure 6D). Furthermore, homologous 18-bp DNA sequences were identified in the upstream regions of *gylR* and *glpF1* homologs in all the *Streptomyces* genomes harboring a *glp* operon (Supplementary Sheet 3). Analysis of these 18-bp DNA sequences led to the identification of an 18-bp sequence motif (Figure 6E). These results elucidated the detailed DNA binding sequences of GylR in *S. clavuligerus* NRRL 3585. Moreover, a core 18-bp sequence motif was identified to be bound by GylR-like regulators in *Streptomyces* based on experimental data and bioinformatic analysis. The molecular regulatory mechanism of GylR revealed by our study and previous reports presumably represents a general regulatory mechanism in glycerol metabolism in *Streptomyces*.

## Discussion

Glycerol can be obtained from multiple paths, especially the growing biodiesel industry that produces glycerol as a main byproduct. The expanding production and reducing cost have made glycerol readily available for versatile applications, one of which is to use it as a carbon source for microorganisms to produce value-added products (da Silva et al., 2009; Guo et al., 2013; Poblete-Castro et al., 2020). As the fertile producers of natural product drugs, streptomycetes can metabolize glycerol through the *glp* operon that is negatively regulated by GylR, as evident from previous studies in *S. coelicolor* A3(2) and *S. clavuligerus* NRRL 3585 (Smith and Chater, 1988b; Hindle and Smith, 1994; Baños et al., 2009). In this study, by analyzing all completed genomes of the phylum *Actinobacteria* available in the NCBI Genome database, we found that the *glp* operon-like glycerol utilization gene clusters are conserved in *Streptomyces* and several other genera of *Actinobacteria*, suggesting a common glycerol metabolic pathway in these microbes. Using *S. clavuligerus* NRRL 3585 as a model system, our *in vivo* data further confirmed that GylR represses the expression of *glp* operon and *gylR*, and the inhibition could be relieved upon addition of glycerol to the medium. Characterization of the effector molecules is important for fully understanding the mechanism of a regulatory protein. Prior to our research, there are only few studies on identifying the effectors of GylR-like regulators. In *S. coelicolor* A3(2), G3P was proposed to be an effector molecule of GylR based on *in vivo* study (Seno and Chater, 1983; Seno et al., 1984). In *Mycolicibacterium smegmatis*, *in vitro* data showed that G3P is an effector molecule of the GylR homolog, which positively regulates the *glp* operon-like gene cluster (Bong et al., 2019). In *E. coli* and *Pseudomonas* spp., the GlpR proteins regulating the glycerol utilization gene clusters show no significant sequence similarity to GylR, and G3P was suggested to be an effector molecule of these GlpR proteins (Lin, 1976; Poblete-Castro et al., 2020). However, there is a lack of direct evidences of identifying the effector molecules of GylR-like regulators in *Streptomyces*. Our study provided direct evidences showing that both G3P and DHAP are effector molecules of GylR in *S. clavuligerus* NRRL 3585, while glycerol is not. DHAP is for the first time discovered as an effector molecule of GylR-like regulatory proteins. Previous study in *E. coli* suggested that DHAP might have weak interaction with GlpR (Larson et al., 1987). These findings further supported the feedback activation mechanism in the regulation of glycerol metabolism (Lin, 1976; Baños et al., 2009). Specifically, due to the tight control of GylR, the *glp* operon expressed at a very low level initially, which enabled the slow conversion of glycerol to G3P and DHAP. Once these effector molecules reached to concentrations that could induce the dissociation of GylR from the binding of *glp* operon, the glycerol metabolic pathway was fully activated.

We further revealed the binding sequences of GylR at the promoter regions of *glp* operon and *gylR*, which was not known previously. The results suggested that GylR inhibits the expression of *gylR* and *glp* operon through impeding the transcription initiation and elongation, respectively. Based on experimental data and bioinformatic analysis, we were able to identify a palindromic 18-bp sequence motif important for the binding of GylR-like regulators in *Streptomyces*. It is worth noting that, the *glp* operon in *Streptomyces* could also be regulated by other proteins or pathways. In *S. coelicolor* A3(2), the *glp* operon is negatively regulated by the pleiotropic protein NdgR, the binding site of which does not overlap with the binding sequences of GylR (Lee et al., 2017). In addition, the *glp* operon in *S. coelicolor* A3(2) was reported to be subject to carbon catabolite repression (Smith and Chater, 1988a,b).

Glycerol-inducible regulatory elements have many applications in the metabolic engineering and synthetic biology research fields. The promoter of *glp* operon (P*glp*) has been successfully used to coordinate the glycerol utilization with natural product biosynthesis, leading to 3.2-fold enhanced clavulanic acid production in *S. clavuligerus* NRRL 3585 (Guo et al., 2013). A glycerol-inducible expression system based on the antiterminator protein GlpP and its target promoter PglpD was applied in the heterologous production of aspartase and nattokinase in *B. subtilis* (Han et al., 2022). Replacing the promoters of *zwf*, *pgi* and *pfk1* genes with the glycerol-inducible promoter pGUT1, resulting in significantly improved production of *myo*-inositol in *Pichia pastoris* (Zhang et al., 2022). Understanding the underlying molecular regulatory mechanisms in glycerol metabolism will lay a foundation for the design of efficient and simple glycerol-inducible regulatory tools.

In conclusion, by taking *S. clavuligerus* NRRL 3585 as a model system, we have identified the effector molecules and binding sequences of GylR, a key regulator in the glycerol metabolic pathway. Furthermore, we identified a core 18-bp sequence motif that is minimally required for the binding of GylR-like regulators in *Streptomyces*. These findings further revealed the molecular mechanism mediated by GylR in the regulation of glycerol metabolism. The information is of importance for the design of better glycerol utilization pathways that can improve utilization of glycerol to produce valuable products. Moreover, these findings would facilitate the development and application of glycerol-inducible regulatory elements for synthetic biology research in the future.

## Data availability statement

The original contributions presented in the study are included in the article/Supplementary material, further inquiries can be directed to the corresponding author.

## Author contributions

YZ, KF, and GP conceived and designed the project. CZ and YZ performed the experiments. KF carried out the bioinformatics analyses. CZ, KF, and GP analyzed the data and wrote the manuscript with the input of all authors. YH, WW, and ZL provided key suggestions and revised the manuscript. All authors contributed to the article and approved the submitted version.

## Funding

This work was supported by the National Key R&D Program of China (2021YFC2100600 and 2020YFA0907700), and the National Natural Science Foundation of China (32070067).

## Conflict of interest

The authors declare that the research was conducted in the absence of any commercial or financial relationships that could be construed as a potential conflict of interest.

## Publisher’s note

All claims expressed in this article are solely those of the authors and do not necessarily represent those of their affiliated organizations, or those of the publisher, the editors and the reviewers. Any product that may be evaluated in this article, or claim that may be made by its manufacturer, is not guaranteed or endorsed by the publisher.
