# Supplementary material for: Molecular mechanism of GylR-mediated regulation of glycerol metabolism in Streptomyces clavuligerus NRRL 3585
Source: Front Microbiol. 2022 Nov 25;13:1078293. doi: 10.3389/fmicb.2022.1078293 (PMC9732521; doi:10.3389/fmicb.2022.1078293)
Supplement: Supplementary file 1 [file Data_Sheet_1.pdf]

Supplementary Material

Molecular mechanism of GylR-mediated regulation of glycerol metabolism in *Streptomyces clavuligerus* NRRL 3585

Chaobo Zhang[1,2], Youbao Zhao[1], Zilong Li[1], Weishan Wang[1,2], Ying Huang[1,2], Guohui Pan[1,2], Keqiang Fan[1*]

[1] State Key Laboratory of Microbial Resources, Institute of Microbiology, Chinese Academy of Sciences, Beijing, China

[2] University of Chinese Academy of Sciences, Beijing, China

*** Correspondence:**
Keqiang Fan
fankq@im.ac.cn

1 Supplementary Figures

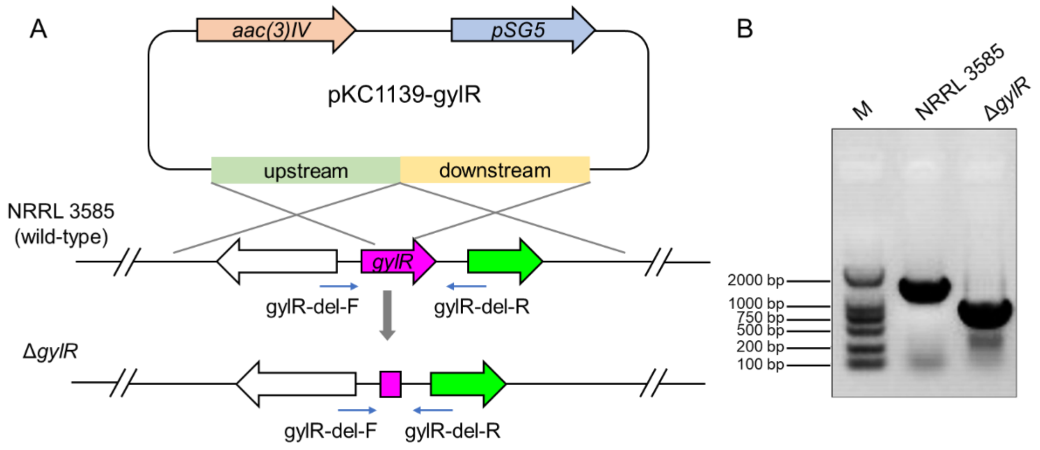

Supplementary Figure 1. Construction of the in-frame deletion mutant strain *S. clavuligerus* Δ*gylR*. (A) Schematic representation for the deletion of *gylR* in the wild-type strain *S. clavuligerus* NRRL 3585 by homologous recombination. The primers gylR-del-F and gylR-del-R were used in the PCR verification of the genotype of Δ*gylR*. (B) PCR verification of the genotype of Δ*gylR*. Lane 1, DNA marker; lane 2, a 1503 bp DNA band was observed in the PCR reaction using the NRRL 3585 genomic DNA as the template; lane 3, a 1044 bp DNA band was observed in the PCR reaction using Δ*gylR* genomic DNA as the template.

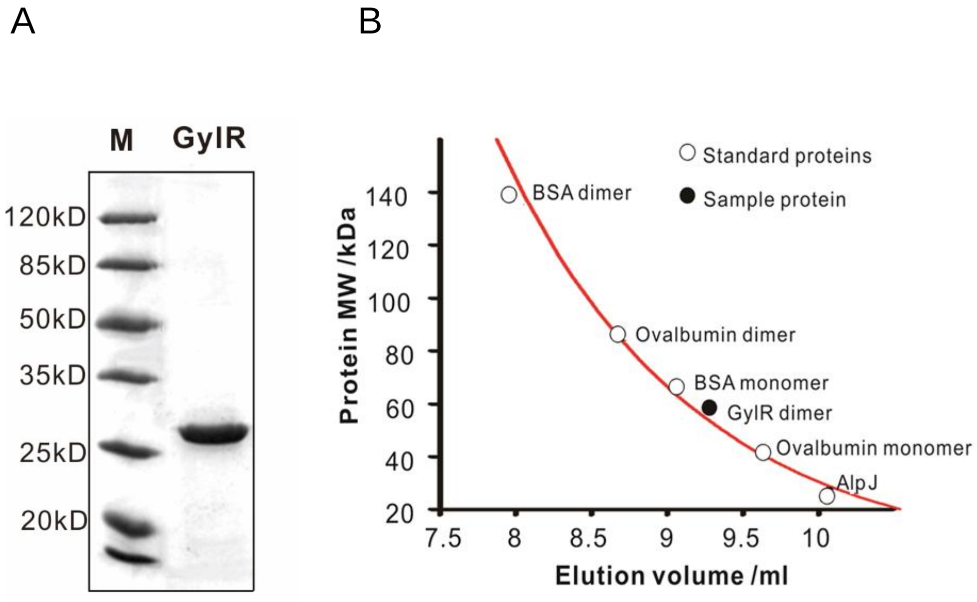

Supplementary Figure 2. Purification of GylR and size-exclusion chromatography analysis. (A) SDS-PAGE analysis of purified GylR. Lane 1, molecular mass markers; lane 2, purified GylR. Samples were separated by 12% SDS-PAGE and stained with Coomassie brilliant blue R-250. (B) GylR forming a dimer according to size-exclusion chromatography analysis. BSA (bovine serum albumin, monomer: 67 kDa, dimer: 134 kDa), ovalbumin (monomer: 43 kDa, dimer: 86 kDa), and AlpJ (27 kDa) (Pan et al, 2017) proteins were used as the standard proteins. The molecular weight of C-terminal His_6-tagged GylR was determined as 56 kDa, showing that it forms a dimer, as the calculated molecular weight of His_6-tagged GylR monomer is 28 kDa.

Supplementary Table 1. Strains and plasmids used in this study

Strains/plasmids	Description [a]	Source
E. coli strains		
DH5α	*E. coli* host for general cloning	Life Technologies
Rosetta (DE3)	*E. coli* host for protein production	Life Technologies
ET12567(pUZ8002)	Methylation-deficient *E. coli* host for intergeneric conjugation, Kan^R, Chl^R	(Kieser et al, 2000)
Streptomyces clavuligerus strains		
NRRL 3585	Wild-type (WT) strain, producer of clavulanic acid	NRRL<br>(Guo et al, 2013)
Δ*gylR*	*gylR* in-frame deletion mutant strain	This study
WT-GFP	*Streptomyces clavuligerus* NRRL 3585 containing plasmid pSET152-GFP, Apr^R	This study
WT-Pglp-GFP	*Streptomyces clavuligerus* NRRL 3585 containing plasmid pSET152-Pglp-GFP, Apr^R	This study
Δ*gylR*-Pglp-GFP	*Streptomyces clavuligerus* Δ*gylR* containing plasmid pSET152-Pglp-GFP, Apr^R	This study
Plasmids		
pET-30a(+)	Plasmid for protein production, Kan^R	Novagen
pET30a-*gylR*	pET30a(+) derived plasmid for production of GylR protein, Kan^R	This study
pKC1139	Plasmid for gene inactivation in *Streptomyces* strains, Apr^R	(Wang et al, 2015)
pKC1139-*gylR*	pKC1139 derived plasmid for inactivation of *gylR*, Apr^R	This study
pSET152	Integrative plasmid for gene expression in *Streptomyces* strains, Apr^R	(Guo et al, 2015)
pSET152-GFP	pSET152 derived plasmid containing the GFP gene, Apr^R	This study
pSET152-Pglp-GFP	pSET152-GFP derived plasmid with insertion of promoter Pglp, Apr^R	This study

[a] Apr^R, apramycin resistance; Kan^R, kanamycin resistance; Chl^R, chloramphenicol resistance.

Supplementary Table 2. Primers used in this study

Primers	Sequences (from 5' to 3') (restriction sites underlined)	Description
gylR-U-F	AGGAAACAGCTATGACATGATTACGAATTCGAGCC GTTGGCGTTGGTGC	Construction of pKC1139-gylR
gylR-U-R	CTGGATCTCGCCAAGGGGACGGGATCCTCTAGAGT CGAGGAGACCTGGGA	Construction of pKC1139-gylR
gylR-D-F	TCTCGCCAAGGGGACGGGATCCTCTAGAGTCGAGG AGACCTGGGA	Construction of pKC1139-gylR
gylR-D-R	TCGTATCCCCACCGACCTCAGCAAGCTTGGCACTG GCCGTCGTTTTACA	Construction of pKC1139-gylR
gylR-del-F	CCCGGGCGATCCGGGCGCCC	Δ*gylR* verification
gylR-del-R	GACGGCAGGGGCCGCGGCTCC	Δ*gylR* verification
RTglpF1-F	GCCCTGGTCGTGGTGGTCTA	RT-qPCR analysis of *glpF1* gene
RTglpF1-R	ATCTCGGTGACGAGGTTCTGC	RT-qPCR analysis of *glpF1* gene
RTglpK1-F	GGCACGCCCGTCAACTCGTA	RT-qPCR analysis of *glpK1* gene
RTglpK1-R	TCCCGCATCCACTGCACCAG	RT-qPCR analysis of *glpK1* gene
RTglpD1-F	GACGAGGCCGCCTTCTCCAT	RT-qPCR analysis of *glpD1* gene
RTglpD1-R	TCACCACGGTCTCCCGCTTG	RT-qPCR analysis of *glpD1* gene
RTgylR-F	CCTGTCGGACATCGCCTCC	RT-qPCR analysis of *glpR* gene
RTgylR-R	AGCTCGGACCGCAGCTCACG	RT-qPCR analysis of *glpR* gene
RThrdB-F	AGGCCCGCACCATCCGTATC	RT-qPCR analysis of *hrdB* gene
RThrdB -R	GGGTCATGTCGAGTTCCTTGGC	RT-qPCR analysis of *hrdB* gene
GFP-F	AAAAACGCGGATCCAAAAAAGGACTAGTAGGAGG ACCCCAATGCGTAAAGGCGAAGAGC	Cloning of GFP gene

GFP-R	AAAAACCG<u>GAATTC</u>TCATCATTTGTACAGTTCATCC ATA	Cloning of GFP gene
Pglp-F	AAAAACGC<u>GGATCC</u>GGGTCGGGCCGCCCGGCC	Cloning of promoter region of *glp* operon
Pglp-R	AAAAAAGG<u>ACTAGT</u>TCCAGGGGAAGGCGAACC	Cloning of promoter region of *glp* operon
gylR-F	AAAAA<u>CATATG</u>GCGAAGAACATCCAGTCGCG	Construction of pET30a-*gylR*
gylR-R	AAAAA<u>CTCGAG</u>GAACCGCCCCGCGCCCAA	Construction of pET30a-*gylR*
BS1-F	CTGGAGCATGGTGCCCATC	Amplification of BS1
BS1-R	GAGGCGATGTCCGACAGGC	Amplification of BS1
BS2-F	GGTGCTCGACCGGTTCCCG	Amplification of BS2
BS2-R	GCCGCCGAGCAGGATCAGA	Amplification of BS2
FAM-gylR	AATGTGCTCCCTGGGATGC	Amplification of labeled BS1
HEX-gylR	TCGACCCTTCCCCACTGC	Amplification of labeled BS1
FAM-glpF1	GGTGCTCGACCGGTTC	Amplification of labeled BS2
HEX-glpF1	TCAGGTGAGGGCGTTG	Amplification of labeled BS2

References

Guo, D., Zhao, Y., Yang, K. (2013). Coordination of glycerol utilization and clavulanic acid biosynthesis to improve clavulanic acid production in *Streptomyces clavuligerus*. *Sci. China Life Sci.*, 56(7):591–600. Doi: 10.1007/s11427-013-4507-z.

Guo, F., Xiang, S., Li, L., Wang, B., Rajasärkkä, J., Gröndahl-Yli-Hannuksela, K., Ai, G., Metsä-Ketelä, M., Yang, K. (2015). Targeted activation of silent natural product biosynthesis pathways by reporter-guided mutant selection. *Metab. Eng.* 28:134–142. Doi: 10.1016/j.ymben.2014.12.006.

Kieser, T., Bibb, M. J., Buttner, M. J., Chater, K. F., Hopwood, D. A. (2000). Practical *Streptomyces* genetics. The John Innes Foundation, Norwich.

Pan, G., Gao, X., Fan, K., Liu, J., Meng, B., Gao, J., Wang, B., Zhang, C., Han, H., Ai, G., Chen, Y., Wu, D., Liu, Z-J., Yang, K. (2017). Structure and function of a C-C bond cleaving oxygenase in atypical angucycline biosynthesis. *ACS Chem. Biol*. 12(1):142-152. Doi: 10.1021/acschembio.6b00621.

Wang, B., Guo, F., Ren, J., Ai, G., Aigle, B., Fan, K., Yang, K. (2015). Identification of Alp1U and Lom6 as epoxy hydrolases and implications for kinamycin and lomaiviticin biosynthesis. *Nat. Commun.* 6:7674. Doi: 10.1038/ncomms8674.
